# Experimentally optimized threading structures of the proton‐coupled folate transporter

**DOI:** 10.1002/2211-5463.12041

**Published:** 2016-02-22

**Authors:** Swapneeta S. Date, Cheng‐Yen Charles Chen, Yidong Chen, Michaela Jansen

**Affiliations:** ^1^ Department of Cell Physiology and Molecular BiophysicsSchool of MedicineTexas Tech University Health Sciences CenterLubbockTXUSA; ^2^ Center for Membrane Protein ResearchSchool of MedicineTexas Tech University Health Sciences CenterLubbockTXUSA; ^3^ Medical Student Summer Research ProgramSchool of MedicineTexas Tech University Health Sciences CenterLubbockTXUSA

**Keywords:** anticancer drug, membrane protein, proton‐coupled folate transporter, structural model, substituted cysteine accessibility method

## Abstract

The proton‐coupled folate transporter (PCFT, SLC46A1) transports folic acid across the plasma membrane, together with an excess of protons such that the net charge translocation is positive. We developed 3D structural models of PCFT threaded onto the X‐ray structures of major facilitator superfamily (MFS) members that were identified as close structural homologues. The model of PCFT threaded onto the glycerol‐3‐phosphate transporter (GlpT) structure is consistent with detailed accessibility studies in the absence of extracellular substrate and at pH 7.4 presented here, and additionally with a multitude of other mutagenesis and functional studies. Characteristic MFS structural features are preserved in this PCFT model, such as 12 transmembrane helices divided into two pseudosymmetric bundles, and a high density of positive charges on the periphery of the cytoplasmic site that allow interactions with negatively charged lipid head‐groups. Under the experimental conditions, PCFT predominantly samples the resting state, which in this case is inward‐open. Several positions lining the substrate cavity have been identified. Motif A, a helix‐turn‐helix motif that is a hallmark of MFS transporters between transmembrane segments II and III is oriented appropriately to interact with residues from transmembrane segments IV as well as XI upon conformational transition to the outward‐open state. A charge‐relay system between three charged residues as well as apposing glycines in two α‐helices, both contributed to by motif A, become engaged when PCFT is modeled on the outward‐open state of a putative proton‐driven transporter (YajR).

**Abbreviations**
GlpT: glycerol‐3‐phosphate transporter
MFS: major facilitator superfamily
MTSEA‐biotin: 2‐((Biotinoyl)amino)ethyl methanethiosulfonate
PCFT: proton‐coupled folate transporter
sulfo‐NHS‐LC‐biotin: Sulfosuccinimidyl 6‐(biotinamido) hexanoate
YajR: putative proton‐driven transporter

Folate cofactors play important roles in more than a hundred metabolic reactions in cells. Folates are required for synthesis of DNA precursors and amino acids 1. Folate deficiency is associated with disorders such as neural tube defects, obesity, anemia, developmental delays, hypercysteinemia, and a resulting increase in the risks for heart diseases 2. Absorption and distribution of folates in the human body is mediated by three folate transport proteins, proton‐coupled folate transporter (PCFT, SLC46A1), reduced folate carrier (RFC, SCL19A1), and folate receptor (FR, FOLR1). The human proton‐coupled folate transporter (*hs*PCFT) is the primary means of absorption of dietary folates in humans 3. PCFT is mainly expressed at the apical membrane in the upper small intestine and to a lesser extent in kidney, placenta, choroid plexus and liver 4. PCFT mediates uphill transport of folates coupled to the downhill transport of protons 3, 5.

Hereditary folate malabsorption (HFM), resulting from point mutations in the gene encoding PCFT manifests in form of blood and CNS folate deficiency 6, 7. Approximately 20 mutations in the PCFT gene have been reported that result in HFM 6, 8, 9, 10, 11, 12, 13, 14, 15, 16. PCFT belongs to the major facilitator superfamily (MFS) of transporters. As is characteristic for MFS transporters, PCFT has 12 transmembrane α‐helices with both the N‐and C‐termini in the cytoplasm 17, 18. PCFT localized to the plasma membrane was found to be monomeric when expressed in *Xenopus laevis* oocytes upon injection of mRNA for PCFT, and also upon stable expression in a CHO‐K1 cell line 19. Upon transient overexpression in HeLa cells total protein samples indicated diverse oligomeric assemblies 4. PCFT, similar to other MFS members, is predicted to follow an ‘alternate access model’ for folate transport in which a central folate‐binding pocket is accessible to only either the cytoplasmic or extracellular side 20, 21, 22.

The substituted cysteine accessibility method (SCAM) was originally developed to identify channel‐lining positions in α‐helical transmembrane segments of the nicotinic acetylcholine receptor, a ligand‐gated ion channel 23. In general, Cys react several orders of magnitude faster with methanethiosulfonate (MTS) reagents when exposed to an aqueous environment as opposed to when embedded in the lipid bilayer. Reactivity is also influenced by local steric and electrostatic factors, as well as pH. SCAM has been extensively applied to numerous membrane transport proteins to study their topology, ligand‐binding sites, and substrate pathways 24, 25, 26, 27, 28, 29, 30. Select positions in PCFT have been studied using SCAM to determine its overall topology and to study positions involved in substrate translocation 14, 18, 31, 32, 33, 34, 35. In the absence of a high‐resolution crystal structure of PCFT, extensive analysis of the aqueous surfaces of PCFT can be used to guide the generation and optimization of threading models of PCFT based on X‐ray structures of related MFS transporters. Detailed solvent‐accessibility studies of the extracellular face of PCFT were previously carried out in our laboratory 35.

In this study, we have determined the cytosolic solvent‐accessibility profile of PCFT engineering 36 single‐Cys, one at a time, in a Cys‐less PCFT background. In all constructs, the Cys substitutes a single endogenous amino acid. Substitutions are indicated as, for example, ‘V111C’, in which the endogenous Valine at position 111 was substituted by a Cys. The accessibility of individual Cys was assessed using the reagent 2‐((biotinoyl)amino)ethyl methanethiosulfonate (MTSEA‐biotin) in the absence and presence of the permeabilizing agent digitonin and normalized using the plasma membrane expression of each construct using sulfosuccinimidyl 6‐(biotinamido) hexanoate (sulfo‐NHS‐LC‐biotin), respectively. The experimentally determined accessibility was then compared to accessibility observed in threading models of PCFT to identify models that reflect the experimental results. Our results indicate that certain segments of select transmembrane α‐helices of PCFT display high solvent accessibility. We predict that these helices outline the folate‐binding pocket of PCFT. A few of these positions have been predicted to interact with folate‐substrate based on mutagenesis studies 20, 36. The helices we identified to interact with the substrate also have helix breaks in the middle of their transmembrane region, which may aid in the proper orientation of folate‐interacting residues. In summary, our accessibility studies identify the loop‐helix boundaries of the cytoplasmic face of PCFT, and further substantiate or identify new folate‐binding pocket positions of PCFT. Importantly, crucial structural features, including the motif A charge‐relay system of MFS transporters between TM II, TM III, TM IV, and TM XI, that are involved in functional conformational transitions are oriented appropriately in our models, thus providing a first step in elucidating the translocation mechanism of PCFT.

## Results

### Optimization of oocyte plasma‐membrane permeabilization

Previously, we investigated solvent accessibility of the extracellular face of PCFT using SCAM 23, 35. We studied 40 positions along the loop‐helix boundaries at the extracellular face of PCFT. Positions where an engineered Cys was covalently modified with a Cys‐specific reagent (MTSEA‐biotin) applied to intact *Xenopus laevis* oocytes as the heterologous expression system were defined as accessible from the extracellular side. All single‐Cys were introduced in a Cys‐less background (PCFT‐CL), in which all seven native Cys were replaced by Ser, that is functionally similar to wild‐type PCFT 18, 35. We also evaluated the experimentally observed accessibility in threading models of PCFT generated by LOMETS (Local Meta‐Threading‐Server) and other threading servers 37. Our PCFT models based on the structure of glycerol‐3‐phosphate transporter (GlpT, PDB: 1PW4) and tripeptide proton symporter (PepT_St_, PDB: 4APS) best fit our extracellular accessibility data. In the present study, we have experimentally determined the solvent‐accessibility profile of the intracellular cytoplasmic face of PCFT. We define a residue as accessible from the intracellular side, if the Cys‐modification occurs in the presence of a permeabilizing agent for the plasma membrane (digitonin), but not in its absence.

**Figure 1:**
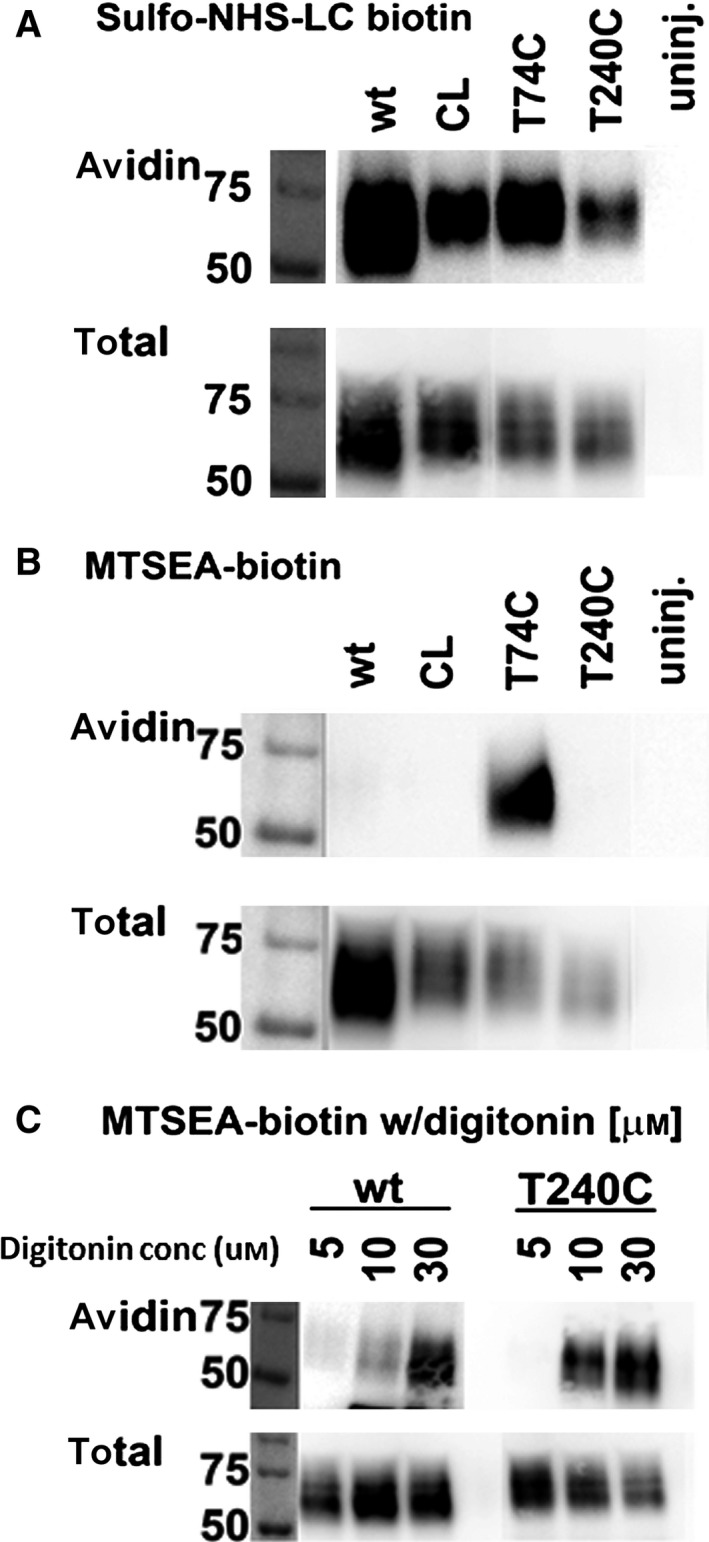
Optimization of permeabilization conditions for labeling of intracellular Cys. Western blot using V5 antibody that recognizes V5 tag engineered at the C‐terminus of PCFT. Oocytes were biotinylated with different reagents as indicated for each panel. Samples of biotinylated proteins isolated with avidin beads (Avidin), or cleared lysates (total) were separated by SDS‐PAGE. (A) Labeling by sulfo‐NHS‐LC‐biotin indicates expression of wild‐type PCFT (wt), Cys‐less‐PCFT (CL) and two single‐Cys PCFT Cys‐substitution constructs, T74C and T240C, at the plasma membranes of *X. leavis* oocytes. Uninjected oocytes (uninj.) were used as negative control. (B) MTSEA‐biotin is membrane impermeant under our experimental conditions and labels accessible extracellular Cys residues. Accessible Cys at the extracellular face of PCFT (T74C), the positive control, is labeled but not the Cys exposed at the solvent accessible cytoplasmic face of PCFT (wt and T240C). (C) Labeling of Cys exposed to the solvent‐accessible cytoplasmic face is achieved by permeabilizing oocytes with 10 and 30 μm digitonin.

Initially, to optimize the plasma membrane permeabilizing conditions in *X. laevis* oocytes, we selected two PCFT single‐Cys constructs, T74C that was shown to be accessible from the extracellular side in MTSEA‐biotin‐labeling experiments, and T240C that was shown to be accessible to MTSEA‐biotin after permeabilization of Hela cells with digitonin, but not in the absence of digitonin 18, 35. Oocytes were injected with RNA coding for wild‐type PCFT (Wt), Cys‐less PCFT (CL), and the two single‐point Cys‐substitution constructs of PCFT‐CL, respectively. Expression of all four PCFT constructs at the oocyte plasma membrane was probed by labeling with sulfo‐NHS‐LC‐biotin. Sulfo‐NHS‐LC‐biotin labels primary amines, primarily Lys sidechains, present in extracellular protein segments. Biotinylated proteins were isolated using avidin beads, separated by SDS‐PAGE, blotted to PVDF membranes, and detected with an antibody against a V5 tag engineered at the C‐terminus of PCFT. Total protein fractions indicated that all four constructs, Wt‐PCFT, CL‐PCFT, T74C, and T240C, were translated (Fig. 1A,B,C, total), and the sulfo‐NHS‐LC‐biotin fraction indicated that all four expressed at the plasma membrane (Fig. 1A, avidin). MTSEA‐biotin is membrane impermeant under our experimental conditions. This is evident from the absence of biotinylation of the T240C construct without permeabilization of *Xenopus* oocytes, whereas biotinylation is observed for T74C, in which the introduced Cys is present in the first extracellular loop of PCFT (Fig. 1B, avidin). Wt‐PCFT contains seven Cys residues. Only two Cys residues are located in extracellular loops and are linked by a disulfide bond, hence no biotinylation of these two Cys residues is seen by MTSEA‐biotin 5, 18. One Cys residue is located at the loop‐helix boundary of the fourth intracellular loop of PCFT and is accessible to MTSEA‐biotin when cells are permeabilized with digitonin. The other endogenous Cys are predicted to reside in the transmembrane region. To optimize conditions for digitonin to permeabilize *X. laevis* oocyte plasma membranes to MTSEA‐biotin, three different concentrations of digitonin were compared, 5 μm, 10 μm, and 30 μm 38, 39. After a 30‐min treatment, no labeling of T240C and wt‐PCFT was observed at a 5‐μm digitonin concentration, whereas significant labeling for both was observed at 10‐ and 30‐μm digitonin concentrations (Fig. 1C, avidin). A concentration of 10 μm digitonin was subsequently used as the concentration to permeabilize *X. laevis* oocytes for the accessibility studies.

### Selection and generation of single‐Cys‐substitution constructs

**Figure 2:**
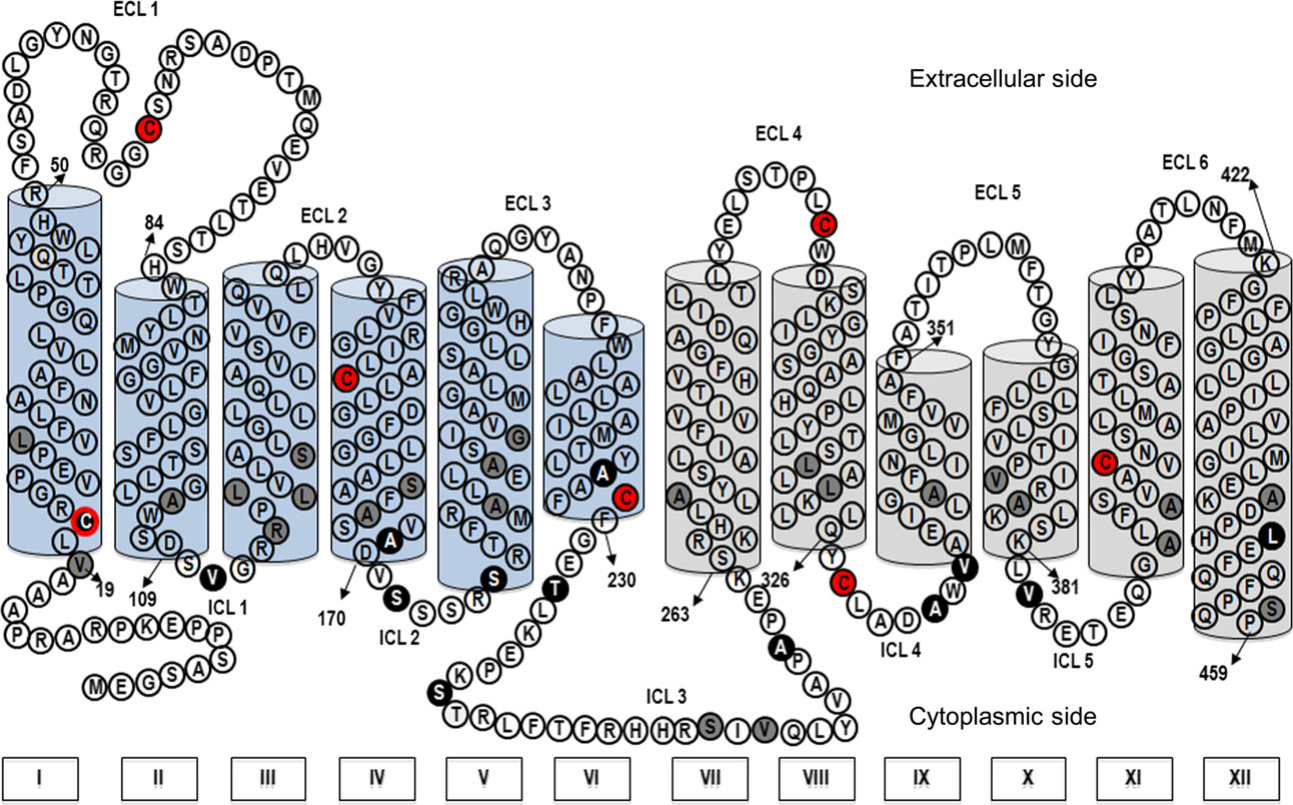
Selection of positions for the accessibility analysis of the cytoplasmic face of PCFT. A 2‐dimensional (2D) reference model of PCFT developed based of the crystal structure of glycerol‐3‐phosphate transporter (GlpT, PDB: 1PW4). The cylindrical structures represent transmembrane segments (TM) from the N‐terminal (blue cylinders) and C‐terminal (gray cylinders) halves of PCFT. Red‐filled circles indicate the seven native Cys mutated to Ser in CL‐PCFT. 13 positions from the cytoplasmic face of PCFT were initially probed to determine their solvent accessibility (white letters in black‐filled circles). Additionally, 23 positions mostly predicted to be deeper within the transmembrane region (black letter in gray‐filled circles) were investigated.

Amino acids for which mutation to Cys is expected to be less disruptive for folding and/or functionality were preferentially selected. Small hydrophobic amino acids, hydroxyl or amide side chains (VMILSTHNGQA) were preferred over charged and aromatic residues (DERKFYW) or proline. Based on the accessibility studies of the extracellular face of PCFT 35, we identified glycerol‐3‐phosphate transporter (GlpT, PDB: 1PW4) and tripeptide proton symporter (PepT_St_, PDB: 4APS) as good initial templates for modeling PCFT. The PCFT 2D‐model used in the current study to guide selection of positions to investigate for accessibility scanning is developed based on the crystal structure of GlpT (Fig. 2).

### Initial accessibility screen of 13 single‐Cys PCFT substitution constructs

Proton‐coupled folate transporter is predicted to contain 12 α‐helical transmembrane segments, as is a key feature for MFS transporters. For each of these 12 transmembrane helices we initially selected at least a single position – or for longer loops more positions – located in a predicted intracellular loop or loop‐helix boundary. Cys at 13 positions were such probed for their accessibility toward MTSEA‐biotin (Fig. 2, black‐filled circles).

Since mutations may alter plasma membrane expression levels, plasma membrane expression of all single‐Cys PCFT constructs in *X. laevis* oocytes was assessed using surface biotinylation with sulfo‐NHS‐LC‐biotin. In every set of experiments, uninjected oocytes, wt‐PCFT‐injected, and CL‐PCFT‐injected oocytes were used as controls. Since the amount of a given construct expressed on the plasma membrane will affect how much can be biotinylated not only by sulfo‐NHS‐LC‐biotin but also MTSEA‐biotin, we normalized the accessibilities of all constructs by calculating the ratio between the MTSEA‐biotin‐labeling intensity in the presence of digitonin (MTSEA‐biotin w/digitonin) divided by the sulfo‐NHS‐LC‐biotin‐labeling intensity (Fig. 6).

**Figure 3:**
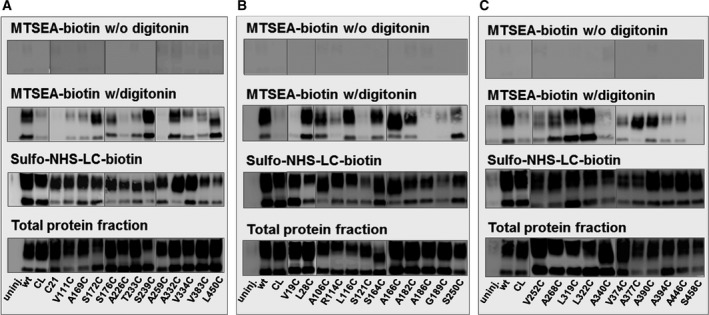
Western Blotting to determine accessibility of single‐Cys PCFT constructs. Individual positions were probed by substituting individual residues to Cys (first letter and number indicates the native amino acid and position that was substituted to Cys; V111C is Valine 111 > Cys), one at a time. Oocytes injected with respective mRNAs were divided and labeled with MTSEA‐biotin in the absence of digitonin; with MTSEA‐biotin in the presence of 10 μm digitonin, as a permeabilizing agent; with sulfo‐NHS‐LC‐biotin to ascertain and quantify expression of Cys‐substitution constructs at the plasma membrane. Labeled proteins were isolated with avidin beads; nonavidin‐bead purified fraction represents total PCFT translated in oocytes. Proteins were separated by SDS‐ PAGE, transferred to PVDF, and probed with V5 HRP antibody. All constructs were translated and expressed in total protein fractions and at the plasma membrane. Gray boxes delineate different gels. Wild‐type‐PCFT (wt), uninjected PCFT (uninj.), and Cysless PCFT (CL) were included in every set of the experiment as controls. (A) Initial screen of Cys at positions expected to be within intracellular loops or loop‐helix boundaries, (B) accessibility within the N‐terminal half, helices I‐VI, (C) Accessibility within the C‐terminal half, helices VII‐XII.

**Figure 4:**
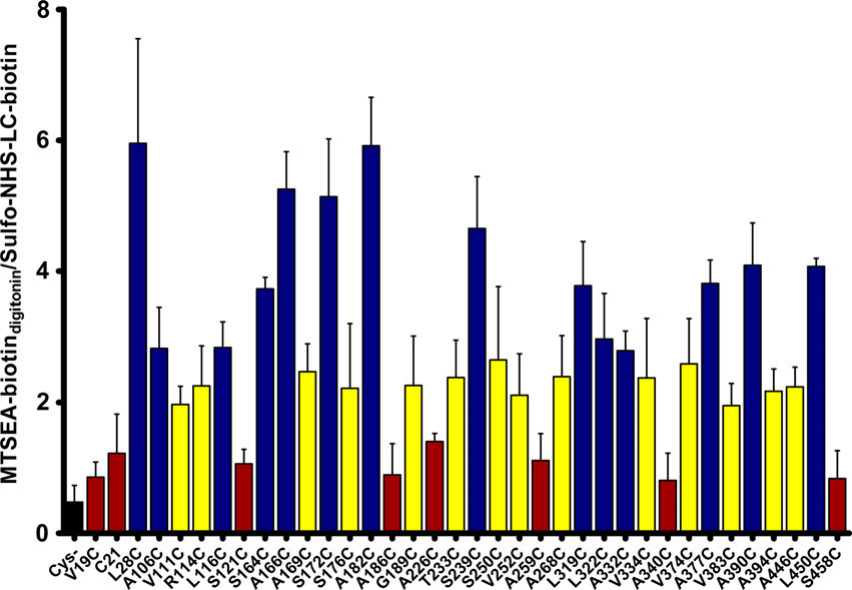
Statistical analysis of accessibility data. For each Cys‐substitution construct we calculated the normalized accessibility by dividing MTSEA‐biotin‐labeling intensity in the presence of digitonin by sulfo‐NHS‐LC‐biotin‐labeling intensity. The resulting data were analyzed with One‐way ANOVA and Dunnett's multiple comparison tests against PCFT‐CL (Cys‐). Constructs that are not significantly different from PCFT‐CL (control) were identified as inaccessible (*P* ≥ 0.05, red), constructs that were significantly different from PCFT‐CL were identified as moderately accessible (0.0001 ≤ *P* ≤ 0.05, yellow), or accessible (*P* < 0.0001, blue). The bars represent standard error of the mean.

All 13 constructs of the initial screen expressed in total and plasma membrane protein fractions (Fig. 3A). For none of the constructs biotinylation with MTSEA‐biotin was observed in the absence of the permeabilizing agent digitonin. Based on the accessibility ratios (Fig. 4), positions S172C, S239C, and A332C are highly accessible to MTSEA‐biotin (Fig. 3A, MTSEA‐biotin w/digitonin and Fig. 6). Based on the initially predicted PCFT structure (Fig. 2), these positions are in intracellular loop (ICL) 2, ICL 3, and ICL 4, respectively. Positions C21 (a native Cys) and A226C are located in predicted helical regions, near the loop‐helix boundary of TM I and VI, respectively, and are inaccessible to MTSEA‐biotin. Positions V111C (TM II), A169C (TM III), S176C (TM IV), V334C (TM IX), and V383C (TM X) located at loop‐helix boundaries are moderately solvent accessible. Residues located within the ca. 30 amino acid‐long cytoplasmic loop in between PCFT TM VI and VII showed varying degrees of accessibility. Position T233C located in the loop near the loop‐helix boundary of TM VI was moderately accessible, position A259C located in the loop near the loop‐helix boundary of TM VII was inaccessible, and position S239C was accessible. The loop between TM VI and TM VII may thus be in part tightly packed with other loops or TM regions. Position L450C is a highly accessible position and based on the predicted structure, this position is located toward the intracellular end of TM XII, which is a long helix formed by 34 amino acid residues.

### Extension of solvent‐accessibility screening with 23 additional positions

We investigated single‐Cys engineered at 23 additional positions from near the cytosolic loop‐helix boundaries and also within the lower half of transmembrane segments (Fig. 2, black letters in gray‐filled circles). The accessibility is discussed for each 3‐helix repeat domain and quantified in Fig. 4. MFS transporters consists out of structurally homologous halves, the N and C domain, that each contains two internal structural repeats that are related by a 180° rotation around an axis parallel to the membrane 40, 41.

In helical bundle I–III, positions V19C (Fig. 3B) and C21 (Fig. 3A) located in proximity to the TM I loop‐helix boundary were inaccessible, whereas position L28C deeper within TM I was highly accessible (Fig. 3B). Positions V111C and R114C located in the loop between TM II and III were moderately accessible. Interestingly, positions A106C and L116C, located higher up in TM II and III, respectively, from the cytoplasmic side, were highly solvent accessible. Position S121C, located five amino acids above highly accessible position L116C, however, was inaccessible (Fig. 3B).

From helical bundle IV–VI, positions A169C and S176C are located at the loop‐helix boundaries in TM IV and V, respectively, and were moderately solvent accessible (Fig. 3A). Positions S164C, A166C, and A182C located higher in TM IV and V, were highly solvent accessible (Fig. 3B). Position G189C located in middle of TM V was moderately solvent accessible, but position A186C, located 3 amino acids below position G189C is inaccessible. This pattern of higher solvent accessibility deeper in a transmembrane segment was also observed in TM I where position L28C deeper in the TM helix was highly solvent accessible, whereas positions lower to L28C, V19C and C21, were inaccessible. This pattern of solvent accessibility within segments of transmembrane helices that are within the depth of the lipid bilayer is to be expected and explained if these helices line the substrate‐binding pocket of PCFT. Residues lining the substrate‐binding pocket and oriented with their side‐chain protruding into the cavity would react with MTSEA‐biotin, whereas residues facing away from the cavity would not.

A total of five positions were studied from the long loop linking the two homologous N and C domains, the TM VI–VII linker. The first half of this loop harbored one moderately accessible position, T233C, and one highly accessible position, S239C (Fig. 3A). The second half of the loop harbored two moderately accessible positions, S250C and V252C (Fig. 3B,C). A259C located at the end of the loop near TM VII, however, was inaccessible (Fig. 3A). This differential accessibility pattern of this long cytoplasmic loop indicates that parts of this loop are tightly packed. The so‐far crystallized MFS transporters display significant structural heterogeneity in their intracellular and extracellular loop regions, ranging from a disordered long TM VI–VII loop in GlpT, PepT, PiPT, and POT 42, 43, 44, 45 to multi‐α‐helical arrangements in XylE 46, indicating that diverse structures in these accessory domains can support similar translocation mechanisms.

The helical bundle of TM VII–IX forms another moderately solvent accessible patch of the cytoplasmic face of PCFT. Position A268C located in TM VII was moderately solvent accessible. Positions L319C and L322C located in TM VIII were highly solvent accessible. Position A340C, located in TM IX was solvent inaccessible (Fig. 3C).

Eight positions were probed from the cytoplasmic face of TM X to XII. Position V383C, located in the small loop between TM X and XI was moderately accessible (Fig. 3A). Position S458C the last but one position before the C‐terminus of PCFT was inaccessible (Fig. 3C). Position V374C from TM X was moderately accessible and position A377C, two amino acids down toward the cytoplasmic side in TM X was highly accessible. Positions A394C and A446C from TM XI and XII, respectively, were moderately solvent accessible and positions A390C and L450C, located one helical turn below A394C and A446C, respectively, toward cytoplasmic side were highly solvent accessible (Fig. 3C).

### Threading structures of PCFT

X‐ray structures of MFS proteins have been solved in diverse conformations. Inward‐open (open to the cytoplasmic side) conformations have been reported for glucose‐proton symporter of *Staphylococcus epidermidis* (GlcP_Se_) 47, lactose permease of *Escherichia coli* (*E. coli)* (LacY) 48, glycerol‐3‐phosphate transporter from *E. coli* (GlpT) 43, oligopeptide‐proton symporter from *Streptococcus thermophilus* (PepT_St_) 45, and the nitrate/nitrite exchanger from *E. coli* (NarK) 49. Outward‐open (open to the periplasm) structures have been observed for the *E. coli* fucose transporter (FucP) 50, and a putative proton‐driven transporter (YajR) 51. Occluded inward or outward conformations with a clam‐shell‐like arrangement around the substrate‐binding site that is buried in the membrane were described for melibiose‐sodium symporter of *Salmonella typhimurium* (MelB_St_) 52, a eukaryotic phosphate transporter (PiPT) 42, *E. coli* xylose transporter (XylE) 46, multidrug transporter (EmrD) 53, and nitrate transporter (NarU) 54. The mere existence of occluded states with compact helix arrangements indicates that a simple ‘rocker‐switch’ mechanism with two banana‐shaped rigid bodies pivoting around the substrate‐binding site constitutes an oversimplification.

**Figure 5:**
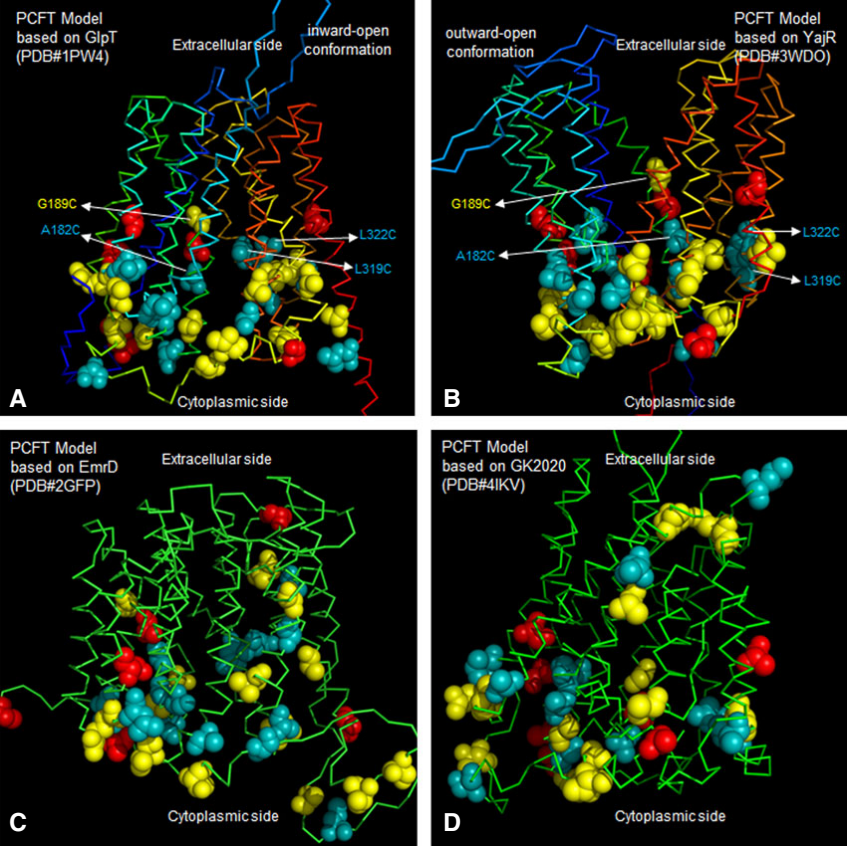
PCFT models. (A) PCFT model in inward‐open conformation based on GlpT. (B) PCFT threaded onto the YajR structure in outward‐open conformation. Side‐chain spheres represent accessibility data color‐coded as accessible (blue), inaccessible (red) and moderately accessible (yellow). Labeling of certain positions within the transmembrane helices of PCFT (A182C and G189C in TM IV, L319C and L322C in TM VIII) was observed in the presence of the permeabilizing agent digitonin, but not in its absence. Note, that in the inward‐open model highly accessible positions (A182C, L322C, and L319C) are located deep within transmembrane segments where their sidechains line the open cavity. The same positions are tightly packed in the outward‐open state. This indicates that PCFT was sampled predominantly in an inward‐open conformation under our experimental conditions (pH 7.4 and no folate). Labeling of such TMs by hydrophilic reagent also indicates their positioning close to the solvent‐accessible folate‐binding pocket of PCFT. (C and D) PCFT models contradicting the experimentally observed accessibility.

The top ten structural homologues with experimentally determined X‐ray structures identified for human PCFT by LOMETS are MFS members. Threading models for PCFT based on these MFS proteins in all three conformational classes, inward‐open, outward‐open, and occluded were generated (Table 1). To assess these models, we visualized positions investigated by SCAM in each model by differential color‐coding of inaccessible, accessible, and highly accessible residues as determined by MTSEA‐biotin labeling in the presence of digitonin (Figs 4 and 5). Based on the accessibility of several residues located within the lower halves of multiple TMs as described above, inward‐facing models in general provide a better representation of the observed PCFT accessibility. From among the inward‐open models of PCFT, the models based on GlpT and GlcP_Se_ (PDB: 4LDS) best fit our experimentally determined accessibility. Figure 5 depicts an inward‐open PCFT model developed based on the crystal structure of GlpT (PDB: 1PW4) and outward‐open model based on YajR (PDB: 3WDO). In the outward‐open YajR model several highly accessible residues deeper in TM segments are in a now‐closed intracellular cavity and therefore not accessible in the model. Therefore, the inward‐open models in which these residues line an intracellular water‐filled cavity are in better agreement with the experimentally determined accessibility. Models contradicting the experimentally observed accessibility were excluded from further analysis; for example if an experimentally inaccessible position was located on an extracellular exposed loop and several other positions did not agree between model and experimental result (Fig. 5C,D).

**Table 1:** Threading templates for PCFT: LOMETS meta server was used to identify closest homologues of PCFT. The conformation of the template X‐ray structures is indicated as inward‐open (IO), outward‐open (OO), or occluded (OC)

	Template protein	PDB	Conformation	*Z*‐score	% Ident.	Modeling server	Release date
GlpT	*E. coli* Glycerol‐3‐phosphate transporter	1PW4	IO	8.9	13	MUSTER	Aug 2003
YajR	*E. coli* YajR Drug efflux transporter	3WDO	OO	11.8	15	MUSTER	Sep 2013
GlcP_Se_	*Staph. epid*. Glucose Transporter	4LDS	IO	20.2	14	SP3	Oct 2013
FucP	*E. coli* Fucose Transporter	3O7P	OO	24.1	11	FFAS	Oct 2010
PepT_St_	*Strep. therm*. Peptide Transporter	4APS	IO	11.7	15	SPARKS‐X	Aug 2012
EmrD	*E. coli* Multidrug transporter	2GFP	OC	7.4	14	MUSTER	May 2006
LacY	*E. coli* Lactose Permease	1PV6	IO	12.8	13	wdPPAS	Aug 2003

### Two‐dimensional PCFT model

**Figure 6:**
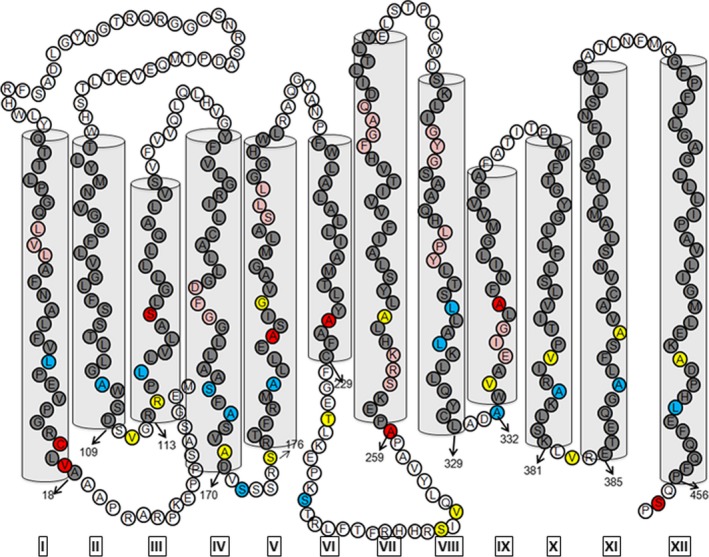
Topology map of PCFT. A topology map of PCFT was developed based on the glycerol‐3‐phosphate transporter, GlpT‐ (PDB: 1PW4) based PCFT model. Gray‐filled circles and cylinders represent PCFT transmembrane helices. Accessibility data are color‐coded as inaccessible (red), accessible (blue), and moderately accessible (yellow). Beige‐filled circles represent helix breaks in the transmembrane regions of PCFT. The presence of TM helix breaks corresponds with higher TM accessibility for helices I, IV, V, VIII, X and XI, supporting the hypothesis that these helices line the folate‐binding pocket of PCFT.

For visualization purposes we generated a topology model of PCFT matched to the threading 3D structure based on the GlpT structure developed through HHSearch (Fig. 6). The accessibility results and potential helix breaks were then mapped onto this model.

## Discussion

This study describes, for the first time, the experimental determination and development of a detailed accessibility profile of the cytoplasmic face of PCFT expressed in plasma membranes. We used SCAM to establish loop‐helix boundaries at the cytoplasmic face of PCFT. We also probed positions higher in TMs from the cytoplasmic side.

Based on the demonstrated reactivity of residues buried within TMs we have identified several TMs as lining the substrate cavity. Characteristic to MFS transporters, a large aqueous pocket at the center of the membrane, a substrate‐binding pocket, is alternatively open to the cytoplasmic or extracellular space 55, 56. Thus, substrate‐translocation cavity residues would have highly accessible transmembrane residues. In addition, upon closer inspection, several helices of the GlpT‐based PCFT threading model are discontinuous, that is, they contain brief helix breaks (Fig. 6). In TM I position L28C that is buried roughly one‐third into the membrane‐bilayer from the cytosolic side is accessible, whereas both investigated more cytosolic positions, V19C and C21, located in close proximity to the loop‐helix boundary, are inaccessible. As indicated in the 2D model (Fig. 6) TM I contains a helix break two helical turns above position L28. This helix break is located near charged and aromatic amino acids positions. Such helix breaks may provide proper orientation for these charged or aromatic amino acids to interact with folate substrate. Discontinuous helices that may allow or mediate conformational changes during substrate translocation 57 have been previously observed in several X‐ray structures of MFS proteins, for example in EmrD 53, XylE 46, GlcP_Sc_ 47, and in MelB_St_ 52. Helix I thus forms a part of the PCFT substrate‐translocation pathway, with L28 facing toward the substrate pocket and V19 and C21 facing away from the pocket during MTSEA‐biotin labeling.

Similarly, in the middle of TM V, position G189 disrupts the helix in a region rich with charged and aromatic amino acids, potential folate‐binding residues. Both A182C and G189C, located on the same face of the TM V α‐helix, were labeled with MTSEA‐biotin, which additionally corroborates the proximity of TM V to the substrate‐binding site. Position 186C faces the opposite site of the helix and is inaccessible. Previously, I188, located in TM V was predicted to be involved in direct interaction with folates 36, and E185 was proposed to be involved in proton coupling 20. TM V is thus another helix near the substrate‐binding pocket. TM IV also harbors highly accessible transmembrane residues and a helix break within a patch of charged and aromatic amino acids. Five residues from the TM IV (147, 152, 157, 158, and 161) have been implicated in interaction with folate substrate 58 and D156 was shown to be important for PCFT structure 13. Together, our data and previous studies thus support that TM IV is also lining the substrate‐binding pocket of PCFT. TM VIII contains two highly accessible positions as well as two helix breaks near charged and aromatic amino acids. Interestingly, our accessibility studies of the extracellular face of PCFT indicated that two highly accessible positions are located in TM VIII near the extracellular end of this helix, L303 and G307 35. TM VIII harbors position Y315 which is important for PCFT function and its biotinylation was blocked in the presence of PCFT substrate 31. TM VIII is thus another helix near the folate‐binding translocation pathway.

**Figure 7:**
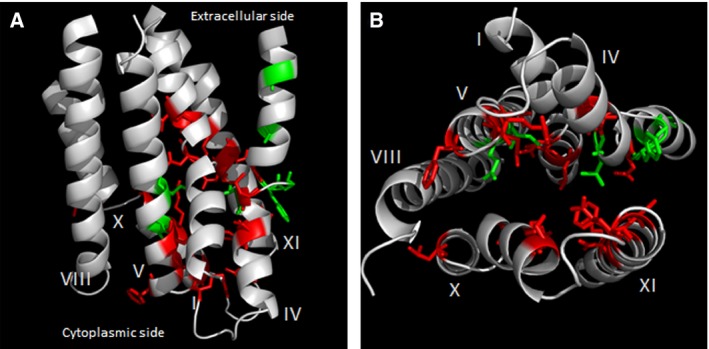
Substrate‐translocation pathway‐lining helices of PCFT. (A) Helices are shown as gray ribbons in a view perpendicular to the membrane. Only the helices involved in shaping the walls of the translocation cavities are shown for clarity. (B) Binding‐site helices viewed from the cytoplasmic face of PCFT. Stick representations of residues shown to be important for PCFT structure‐function in previous studies (green), and those that we predict to be important for folate‐interaction (red). Other PCFT helices are omitted for clarity.

Similar transmembrane solvent‐accessibility observations are made in helices X and XI. Therefore, helices I, IV, V, VIII, X, and XI form a group of helices termed ‘the substrate‐cavity‐lining helices’ (Fig. 7). Certain residues from helices IV and V, as discussed above, have been shown to be important for folate‐PCFT interaction. Our data predict that in addition to TM IV and V, several other helices, TM I, VIII, X, and XI, also harbor residues involved in direct interaction with folate substrates (Fig. 7, red).

In case of TM XII, position A446C and L450C located toward the cytoplasmic side of TM XII were moderately accessible and highly accessible from the cytoplasmic side, respectively. Based on our ligand‐docking studies, helix XII is situated away from the substrate‐binding pocket of PCFT. Moderate solvent‐accessibility of residues located towards the cytoplasmic face may arise by loose packing of helices around this region. Helix XII is a long helix formed by 35 amino acids. In extracellular accessibility studies 35, position F424C, located in TM XII toward the extracellular side, was shown to be highly accessible from the extracellular side. This, thus, indicates loose packing or no packing of this long helix both at the cytoplasmic side and extracellular side.

We plotted our data on PCFT models based on high‐resolution crystal structures of homologues identified with LOMETS (Table 1). All identified template proteins are MFS transporters. Based on the agreement of models to our accessibility data we identified GlpT (PDB: 1PW4) and GlcP_Se_ (PDB: 4LDS) as the best templates for modeling of PCFT. Through multiple sequence alignment and optimization we generated a model of PCFT displaying 38% sequence similarity with the transmembrane region of 4LDS (Table 2). We plotted our accessibility data on both the inward‐ and outward‐facing models of PCFT, as well as occluded conformations (Table 1). The experimental accessibility data is best fit to the inward‐open model of PCFT (Fig. 5). MTSEA‐biotin only labeled positions from the cytoplasmic face when digitonin was present, but not in its absence. Some of these positions are within helices that line a putative hydrophilic substrate‐binding site. Their accessibility from only the cytoplasmic side favors an inward‐facing preferential conformation at the tested conditions (pH 7.4 and no substrate). A transport cycle proposed for LacY indicates a formation of a deprotonated apo‐intermediate state where LacY is present in an inward‐open conformation with neither substrate nor protons bound.

**Table 2:** Transmembrane regions display higher conservancy. For the threading structures the % identity of the entire PCFT sequence, as well as both the % identity and % similarity of the transmembrane region only were calculated

	Template protein	PDB	%Ident.	TM only % Ident.	TM only% Simil.
GlpT	*E. coli* Glycerol‐3‐phosphate transporter	1PW4	13	18	28
GlcP_Se_	*Staph. epid*. Glucose Transporter	4LDS	14	22	38

**Figure 8:**
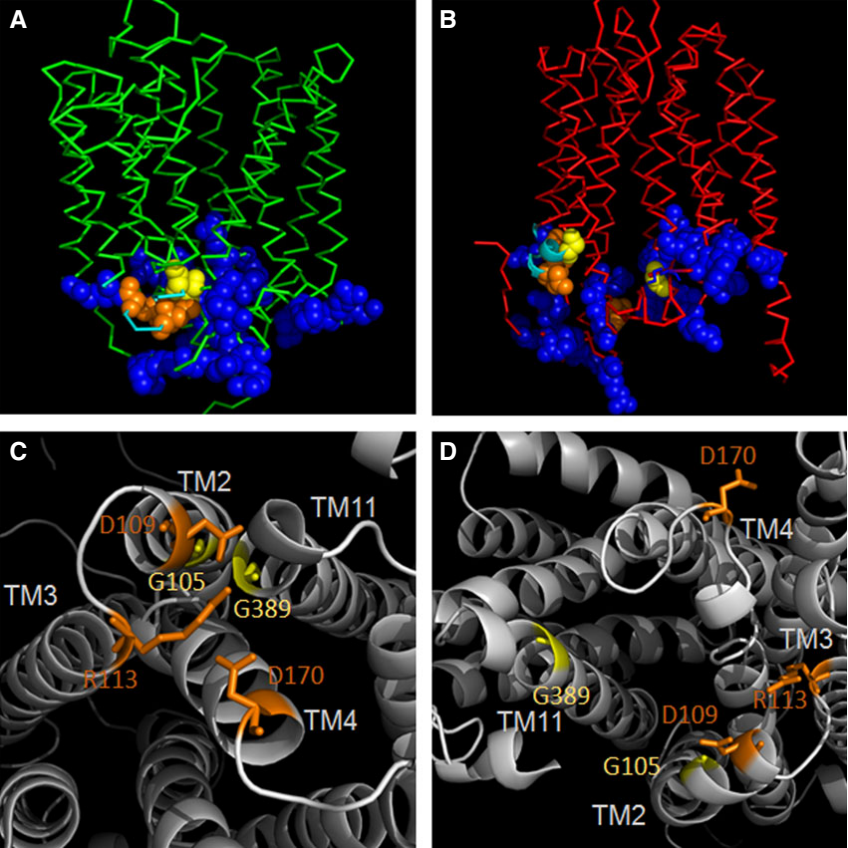
Arrangement of the highly conserved MSF motif A in outward‐ and inward‐open conformations. (A) Ribbon representation of the model of PCFT based on the crystal structure of YajR (PDB: 3WDO) (green) in outward‐open conformation. (B) PCFT model based on GlpT (PDB: 1PW4) (red) in inward‐open conformation. Blue spheres represent positively charged residues. This high density of positive charges is in agreement with the MFS characteristic ‘positive inside’ rule. Yellow spheres represent two glycines, G105 and G389, that are predicted to engage in close helix–helix contacts in the outward‐open conformation of PCFT. Orange spheres represent the charge‐relay system D109‐R113‐D170 important for stabilization of the outward‐open conformation of PCFT. (C) and (D) are cytoplasmic views for (A) and (B), respectively, depicting the detailed arrangement of motif A residues in outward‐open and inward‐open conformations, respectively.

To further validate our models, we have studied orientation of characteristic MFS motifs in PCFT. All MFS transporters share a common motif A, a highly conserved motif found in the cytoplasmic loop between TMs II and III 59. Because of the internal symmetry duplications of motif A have been identified and described 51, 59. Recently, a transport mechanism was suggested based on the conserved motif A with the help of the X‐ray structure of the proton‐driven transporter YajR. In the outward‐open conformation of YajR, interacting partners of motif A, G^(+1)^xlaD^(+5)^ rxGR^(+9)^kp, could be indentified 51. In PCFT motif A is present as **G** ^105^AWS**D**SV**GR**RP (Fig. 8). G69, G^(+1)^, in YajR forms a close helix–helix contact with conserved Gly residues in TM XI, G337 and G341, and this interaction is essential for the formation of the outward‐open conformation. Glycines play crucial roles in polytopic membrane protein transmembrane helix packing by providing molecular notches at which helices cross 51, 60. In our models of PCFT, G105 from TM II closely apposes G389 from TM XI in the outward‐open model based on YajR, whereas these two residues are widely separated in the inward‐open conformation based on GlpT or GlcP_Se_ (Fig. 8), similar to what has been observed for YajR. D73, D^(+5)^, in YajR is completely buried in the domain interface, and tightly packed, so that there is virtually no solvent accessible surface of its side‐chain in the outward‐open conformation of YajR; the same residue becomes solvent‐accessible in the inward‐open conformation. In case of PCFT, the region of motif A is moderate to highly solvent‐accessible under our experimental conditions. This is another indication that an inward‐open conformation of PCFT is sampled predominantly during Cys labeling. In YajR, positively charged R77 located in the TM II to TM III loop interacts with the negatively charged side‐chains of TM II D73 and TM IV D126. Thereby, the three residues form a charge‐relay system in the outward‐open conformation. In PCFT, this charge‐relay system may be formed by residues TM II D109 – loop R113 – TM IV D170. The basic amino acid residue at position 113 of PCFT has been shown to be important for function 9, 10. Both D109 and R113 are highly conserved residues. Mutations of D109 were shown to be nonfunctional 13. D170 is also highly conserved across species, and mutation of D170 to Lys interferes with function 13. This indicates that PCFT residues D109‐R113‐D170 are crucial for function, and that they – based on the here‐developed models – may interact to form a charge‐relay system that is crucial for the oscillation of PCFT between inward‐ and outward‐open conformations. A recent study indicated that the loop connecting TM II and TM III is reentrant ‘similar to those reported for other MFS transporters (e.g., EAAT1)’ based on the labeling of positions 105 and 110 with MTSEA‐biotin in the absence of permeabilizing agent 32. While EAAT1 contains a reentrant loop, it is not a member of the MFS transporters. Intriguingly, position 89 that is inaccessible in our hands 35 is accessible in their study. In addition, even wild‐type PCFT reacted with MTSEA biotin under their experimental conditions. As shown by other studies 18, 31 and explained earlier in the current study, PCFT Cys are not labeled by MTSEA‐biotin in Wt PCFT in the absence of either a permeabilizing agent (to allow access to Cys at cytoplasmic face) or a reducing agent (to break extracellular disulfide bond). It is out of the scope of the present manuscript to determine why Wilson *et al*. concluded a reentrant loop to be present in one of the most conserved motifs of MFS transporters for which available X‐ray structures indicate a helix‐turn‐helix but never a reentrant loop motif.

In conclusion, we have developed and experimentally verified threading models of PCFT based on closely related transporters. While flexible loop regions of the models are likely to display conformations different from their actual conformation in PCFT, the reliability of the models in the core region is high, with sequence identities of ca. 20% and sequence similarities of up to 38% for transmembrane segments (Table 2). Additionally, the model based on GlpT does not only explain our experimental results, but also functional studies published by other laboratories. Our studies identify helices I, IV, V, VIII, X, and XI as involved in shaping the substrate‐binding site. We experimentally identify residues lining an inward‐open pocket, indicating that at pH 7.4 PCFT is predominantly occupying an inward‐open conformation. Importantly, our model displays, in appropriate spatial orientation, several features important for function. The highly conserved motif A, consisting of the charge‐relay system linking the TMII‐III loop with both TM II and TM IV, and the Gly‐mediated helix–helix packing between TM II and TM XI, that is crucial for functional conformational transitions in MFS transporters, is in a spatial orientation allowing its mediation of functional conformational transitions.

## Materials and methods

### Reagents

Sulfosuccinimidyl 6‐(biotinamido) hexanoate (sulfo‐NHS‐LC‐biotin) and High Capacity NeutrAvidin Agarose Resin were purchased from Thermo Scientific (Rockford, IL, USA). 2‐((Biotinoyl)amino)ethyl methanethiosulfonate (MTSEA‐biotin) was obtained from Toronto Research Chemicals (Toronto, Ontario, Canada).

### Mutagenesis and mRNA preparation

A Cys‐less human PCFT construct with c‐terminal V5 epitope tag (GKPIPNPLLGLDST) in the pXOON vector (ctV5‐*Hs*PCFT‐CL) was constructed by replacing all seven endogenous Cys residues with Ser using the QuikChange^™^ Lightning Site‐Directed Mutagenesis kit (Agilent, Santa Clara, CA, USA). This construct was used as a template to generate 36 single‐Cys‐substitution constructs by site‐directed mutagenesis using the QuikChange^™^ kit (Agilent). The mutagenesis primers were generated based on the manufacturer's recommendations and sequences are available upon request. A total of 36 residues were mutated to Cys, one at a time, from the predicted cytoplasmic face of PCFT. All mutations were confirmed by DNA sequencing of the entire gene (Genewiz, South Plainfield, NJ, USA).

### *Xenopus laevis* oocytes

Defolliculated *Xenopus laevis* oocytes were purchased from EcoCyte Bioscience, Austin, TX, USA.

### Biotinylation of *Xenopus laevis* oocytes with sulfo‐NHS‐LC‐biotin (labels primary amines)

Four days after injection, oocytes were washed three times with 6 mL of calcium‐free Ringer's buffer, OR‐2 (82.5 mm NaCl, 2 mm KCl, 1 mm MgCl_2_, and 5 mm HEPES; pH adjusted to 7.4 with NaOH). Surface proteins were biotinylated with 0.5 mg/mL sulfo‐NHS‐LC‐biotin for 30 min at room temperature (RT). Then the oocytes were washed three times with 6 mL of calcium free OR‐2 solution. Subsequently, sulfo‐NHS‐LC‐biotin was quenched by incubating the oocytes for 10 min in buffer H (100 mm NaCl, 20 mm Tris, pH 7.4). The oocytes were triturated at 4 °C in 20 μL/oocyte buffer H^++^ (buffer H with 1% Triton X‐100, 0.5% deoxycholate, and 1× HALT protease inhibitor cocktail, Thermo Scientific), solubilized by rotating at 4 °C for 60 min and spun at 21 000 g for 10 min at 4 °C. After carefully removing the debris and yolk, the supernatant was again spun at 21 000 g for 10 min at 4 °C to remove any residual debris and yolk. To isolate biotinylated proteins, the supernatant was incubated with prewashed and buffer H^++^‐equilibrated NeutrAvidin beads by rotating for 2 h at 4 °C. After incubation the beads were spun at 2500 g for 2.5 min at RT and washed four to five times with 1 mL of buffer H^++^, with the last wash supplemented with 2% SDS, to remove unbound proteins. Biotinlyated proteins were eluted from the beads by adding 60 μL of 4× SDS‐sample buffer with DTT, and incubating at 37 °C for 15 min. Samples were loaded on 4–15% Mini‐PROTEAN^®^ TGX^™^ Precast Gels (Bio‐Rad, Hercules, CA, USA), transferred to PVDF membranes, and probed with V5 HRP antibody (Novel by Life Technologies, Carlsbad, CA, USA) (1 : 5000 in 5% milk for 4 h at RT).

### Biotinylation of *Xenopus laevis* oocytes with MTSEA‐biotin (labels sulfhydryls)

Biotinylation with MTSEA‐biotin was performed using a procedure comparable to the one described for primary amine‐biotinylation described above. Oocytes were incubated with 0.5 mm MTSEA‐biotin in the absence of any permeabilizing agent and in a parallel reaction in the presence of digitonin as a permeabilizing agent. Initial trials to optimize the digitonin concentration included 5, 10, and 30 μm digitonin, leading to 10 μm being used for all subsequent experiments. Excess MTSEA‐biotin was removed by washing extensively with OR‐2.

### Statistical analysis of accessibility data

Taking into consideration that individual Cys‐substitution constructs may express to varying extents on the plasma membrane, we normalized SH‐specific labeling with MTSEA‐biotin in the presence of digitonin versus NH2‐specific labeling with sulfo‐NHS‐LC‐biotin. Accessibility was calculated using the band intensities as follows:

$$\mathrm{Accessibility}={\mathrm{MTSEA}-\mathrm{biotin}}_{\mathrm{Digitonin}}/\text{sulfo‐NHS‐LC‐biotin}.$$

One way ANOVA and Dunnett's multiple comparison test (graphpad prism v6.0 software, San Diego, CA, USA) of accessibilities obtained for PCFT‐constructs vs. PCFT‐CL was used to classify residues as inaccessible, moderately accessible, and accessible based on the calculated normalized accessibility. Cys‐substitution constructs that are not significantly different from PCFT‐CL (control) were identified as inaccessible (*P* ≥ 0.05), those that were significantly different from PCFT‐CL were identified as moderately accessible (0.0001 ≤ *P* ≤ 0.05), or accessible (*P* < 0.0001). Many diverse factors such as side‐chain orientation of the amino acids, apparent pK_a_, and steric factors affect the accessibility of introduced Cys to biotinylating agent.

### Homology modeling of PCFT

Local Meta‐Threading‐Server (LOMETS) was used to search for homologues of PCFT with experimentally determined crystal structures in the PDB database and to generate 3D structural models using default parameters 37, 61, 62. Additional models were generated through MODELLER and SWISS_MODEL 63, 64. Models were studied using PyMol and SPDBviewer.

## Author contributions

SSD and MJ designed experiments. SSD, CYCC, and YC conducted the experiments. SSD and MJ constructed threading models and analyzed the data. All authors contributed to writing the manuscript.
